# Cytokines in Relation to Motor Activity in an Acute Psychiatric Population

**DOI:** 10.3389/fpsyt.2019.00920

**Published:** 2019-12-19

**Authors:** Jeanette Brun Larsen, Astrid Kamilla Stunes, Valentina Cabral Iversen, Arne Einar Vaaler, Solveig Klæbo Reitan

**Affiliations:** ^1^ Division of Mental Health Care, St. Olav’s University Hospital, Trondheim, Norway; ^2^ Department of Mental Health, Faculty of Medicine and Health Science, Norwegian University of Science and Technology, Trondheim, Norway; ^3^ Department of Clinical and Molecular Medicine, Faculty of Medicine and Health Science, Norwegian University of Science and Technology, Trondheim, Norway; ^4^ Medical Clinic, St. Olav’s University Hospital, Trondheim, Norway

**Keywords:** cytokines, psychomotor retardation, agitation, depression, psychosis, acute psychiatric care

## Abstract

**Background:** Deviations in motor activity are important clinical features of several psychiatric disorders in an acute state. Immune activity is associated with several psychiatric disorders and may affect motor activity. We aimed to examine the association between immune activity measured as serum levels of cytokines and deviations in motor activity, in an acute psychiatric setting.

**Methods:** Data on motor activity and immune markers were available on 277 patients admitted to an acute psychiatric inpatient department. The degree of increased or decreased motor activity was clinically assessed at admission. Serum concentrations of the following immune markers were measured: interleukin (IL) -1β, IL-4, IL-6, IL-10, tumor necrosis factor (TNF) -α, interferon (IFN) -γ, and transforming growth factor (TGF) -β.

**Results:** Scores of increased motor activity were negatively correlated with IFN-γ (rho = −0.128, p = 0.033) in an acute psychiatric population. There was also a trend towards an association between motor activity and TGF-β (rho = 0.118, p = 0.050). In a multiple-linear-regression model correcting for age, gender, and body-mass index (BMI, kg/m^2^), the association did not remain significant. No significant correlations between motor retardation and circulating cytokines were found.

**Conclusions:** After adjustment for potential confounders our study did not reveal any significant association between cytokines and motor activity. However, there is an indication of increased Th17 and decreased Th1 responses in relation to increased motor activity in line with the few previous reports in the field. The phenomenon however needs further exploration.

## Introduction

Altered motor activity is gaining increased interest within psychiatric research and may be a prominent finding in an acute psychiatric setting (1, 2). Traditionally, increased or abnormal activity is seen in ADHD, tic disorders, affective disorders, anxiety, and schizophrenia (3). Motor symptoms may also characterize different subtypes of unipolar depression and predict treatment response (2, 4).

Evidence supports a role of immune activity in the etiology and pathogenesis of psychiatric disorders (5). Several studies have demonstrated altered systemic levels of cytokines in patients with schizophrenia, bipolar disorder, and unipolar depression compared to healthy controls (6). These cytokine alterations may also be more prominent in an acute psychiatric setting (6).

Immune activity often is classified into different profiles based on effector mechanisms and characterized by a set of cytokines promoting those effector mechanisms. Th1 profile is characterized by cytokines such as interferon (IFN) -γ and tumor necrosis factor (TNF) -α and mediates potent responses to viruses. Th2 profile is characterized by interleukin (IL) -4 and IL-10. Th2 mediates certain B-cell responses (e.g., immunoglobulin-E production) and opposes Th1. Th17 is characterized by cytokines such as IL-17 and TGF-β and mediates other effector mechanism in the immune system (7).

Cytokines may be the factor mediating altered motor activity in certain psychiatric conditions (8). One mechanism by which cytokines may influence motor activity, is through alterations in neural activity and dopamine metabolism in the basal ganglia (9). It is also shown that treatment with cytokines such as IFN-α induces psychomotor retardation and depressive symptoms in patients with hepatitis (10). Finally, an association between motor activity, psychomotor retardation, and cytokines in outpatients with major depression has been described (11). Agitation is an important clinical syndrome consisting of several symptoms and signs, including increased motor activity. In patients with Alzheimer’s disease, a previous study demonstrated that increased IL-1β was associated with agitation (12). However, few previous studies have investigated the association between increased motor activity only and immune markers.

The aim of this study, was to assess the association between circulation levels of cytokines, motor retardation, and increased motor activity in a sample of patients with a variety of severe mental disorders admitted to an acute psychiatric department. Because changes in motor activity are more common symptoms in certain diagnostic groups, unipolar depression and non-affective psychosis were chosen as subgroups.

## Materials and Methods

### Setting and Participants

This cross-sectional study was conducted in the acute psychiatric inpatient wards of St. Olav’s University Hospital, Trondheim, Norway. All acutely admitted inpatients between September 2011 and March 2012 were asked to participate. At the time of inclusion, the psychiatric department served a catchment area of 228.000 inhabitants (≥18 years old) and represented the only psychiatric inpatient acute unit in the area. Of the total 654 admitted patients in the inclusion period, 382 (58.4%) patients were included in the study. The study was approved by the regional committee for ethics (REC Central number 2011/137) and registered at ClinicalTrials.gov (NCT01415323). All patients gave their written informed consent prior to inclusion. The inclusion process was conducted by specialists in clinical psychology or psychiatry in order to secure that all included patients had the mental capacity to give their consent. The study was conducted according to the Declaration of Helsinki.

### Exclusion Criteria

The following exclusion criteria were applied: (1) chronic or ongoing infections, (2) comorbid autoimmune diseases, (3) C-reactive protein (CRP) levels above 35 mg/L, or (4) lack of patient consent. When patients had multiple admissions, we only included the first admission in our analyses.

### Diagnostic Evaluation

Patients were diagnosed according to the International Classification of Diseases-10 (ICD-10) Criteria for Research (13). The diagnoses were set in a consensus meeting in the treatment staff, always including at least two senior psychiatrists of whom one had personally examined the patient. For subgroup analyses, we included patients with non-affective psychosis (ICD-10 F20–29) and unipolar depression (ICD-10 F32 and F33).

### Assessments

Sociodemographic history, comorbid medical conditions, smoking status, substance abuse, and psychiatric symptoms were recorded after an interview by a staff member. In addition, participants were screened in a general medical examination and routine blood tests, including CRP and leukocyte count. Height and weight were measured for calculation of the body mass index (BMI, kg/m^2^).

The degree of motor retardation and increased motor activity was assessed by an experienced clinician using the Symptomatic Organic Mental Disorder Assessment Scale (SOMAS). SOMAS is a 5-item scale developed to assess atypical depressive symptoms. Item B rates the degree of motor retardation, and item C rates the degree of increased motor activity when the patient was most dysthymic during the previous 24 h (14). Both items are modified from the Positive and Negative Syndrome Scale (PANSS), where item B was modified from PANSS item “motor retardation” (general psychopathology scale, item G7), and item C was assessed from PANSS item “hyperactivity” (positive scale, item P4).

For analyses, patients were subdivided into two groups: with or without increased motor activity according SOMAS item C. If the patients were scored as ≥2 on SOMAS item C, they were grouped as motor active. Similarly, patients were separated into the two groups with or without motor retardation according to SOMAS item B. A score on SOMAS item B ≥ 3 was set to group the patients as motor retarded. In order to simplify the interpretation of findings on SOMAS item B, it was reverse-coded. Therefore, a higher score on both SOMAS item B and C would be interpreted as more severe symptoms of motor retardation or increased motor activity. Subgroup analyses were performed on patients with diagnoses non-affective psychosis group and unipolar depression.

### Serum Analyses of Immune Biomarkers

Blood samples were collected on 9 ml serum tubes with SiO2 without gel between 08.00 and 13.00 (median at 10:00) at the first working day after admission. Strict instructions regarding fasting were not given, though most patients would be fasting overnight. Samples were immediately cooled on ice, protected from daylight, and centrifuged within 30 min (15 min, 1,500 g, 4°C). Serum samples were stored at −80°C until further analysis in a registered Biobank (Biobank1, St. Olav’s University Hospital, Trondheim, Norway). The following parameters were analyzed bymultianalyte profiling Milliplex MAP assays: IL-1β, IL-4, IL-6, IL-10, TNF-α, and IFN-γ (Millipore Corporation, Billerica, MA, US). TGF-β1 was measured by a Bio-Plex Pro TGF-β Assay (Biorad Hercules, CA, US). Intra- and interassay coefficients of variance were less than 10%. The range of detected values was IL-1β: 0.06-198.72 pg/ml; IL-4: 0.92-286.01 pg/ml; IL-6: 0.10-576.42 pg/ml; IL-10 0.10-1125-33 pg/ml; TNF-α: 0.70-268.89 pg/ml; IFN-y: 0.04-1529.70 pg/ml; and TGF-β: 13.07-415.61 ng/ml. The number of samples and percentage under the detection limit was as follows: IL-1β 236 (74.2%), IL-4: 242 (76.1%), IL-6: 177 (55.7%), IL-10:177 (55.7%), TNF-α: 11 (3.5%), IFN-y: 68 (21.4%), and TGF-β: 0.

### Statistical Analyses

Statistical analyses were done using SPSS version 24.0 for Windows. The level of significance was set at p ≤ 0.05, and all analyses were two-tailed. Significant findings were adjusted for multiple testing with the Bonferroni correction (α/k where k = the seven tested cytokines giving α/k = 0.007). Data normality was assessed by using a Kolmogorov-Smirnov test. The distribution of all serum cytokines was skewed, and only TGF-β became normally distributed after logarithmic transformation. Descriptive statistics were calculated by using chi-square tests for categorical variables and student’s independent samples t-tests or Mann-Whitney U test (depending on distribution) for continuous variables. The Spearman correlation coefficient was calculated for the relationship between cytokines and SOMAS. Additionally, we examined the difference in cytokine levels between the groups with and without increased motor activity by using student’s independent samples t-test or Mann-Whitney U test if the data were not normally distributed. The same statistical methods were applied when comparing cytokine levels between the groups with and without motor retardation.

## Results

### Sociodemographic, Clinical, and Inflammatory Characteristics of the Sample

Of the total 382 patients included in the study, 24 were excluded due to infection or autoimmune diseases. This left us with 358 patients for whom serum samples were available, and cytokines were analyzed in 318 patients. For 277 of these 318 patients we also had complete measures for altered motor activity (increased or reduced motor activity). The main ICD-10 diagnostic categories in the 277 patients were unipolar depression (22.7%), substance-use disorders (15.9%), schizophrenia (9.7%), bipolar disorder (12.7%), neurotic, stress-related, and somatoform disorders (10.1%), and personality disorders (8.3%), and other diagnoses (20.6%).

The demographic, clinical, and immune data for the total study population, the non-affective psychosis group, and unipolar depression group are given in **Table 1**.

**Table 1:** Demographic and clinical parameters.

	All patients N = 358	Non-affective psychosis N = 48	Unipolar depression N = 73	*p*-value^a^
Age (years), mean ± SD	38.9 ± 14.8	40.1 ± 11.8	40.1 ± 14.9	0.756^b^
Gender (female), N (%)	174 (49)	19 (40)	42 (58)	0.053^c^
Smoking, N (%)	175 (49)^d^	25 (66)	28 (25)	**0.045^c^**
BMI (kg/m^2^), mean ± SD	25.5 ± 5.9^e^	27.6 ± 6.2	24.9 ± 5.4	**0.015^f^**
Higher education (above high school), N (%)	50 (14)	2 (4)	14 (19)	**0.017^c^**
Unemployment (incl. sick leave), N (%)	255 (71)	41 (85)	42 (58)	**0.002^c^**
Alcohol use upon admission, N (%)	99 (28)	9 (19)	15 (21)	0.808^c^
Substance abuse, N (%)	78 (22)	8 (17)	11 (15)	0.813^c^
Motor retardation score^g^, mean ± SD	1.6 ± 0.7	1.5 ± 0.8	1.7 ± 0.9	0.205^f^
Motor activity score^g^, mean ± SD	1.5 ± 0.9	1.6 ± 1.0	1.3 ± 0.5	0.084^f^
Number with blood samples, N (%)	318 (89)	40 (83)	68 (93)	0.088^c^

a Comparison between non-affective psychosis and unipolar depression. Significance with a p-value < 0.05 is indicated in bold text.

b Independent students’ samples t-test.

c Chi-square test.

d Missing 60 (10 within non-affective psychosis group and 11 within unipolar depression group).

e Missing 89 (15 within non-affective psychosis group and 11 within unipolar depression) observed (data not shown).

f Mann-Whitney U test.

g Motor retardation and motor activity were assessed by items form a Symptomatic Organic Mental Disorder Assessment Scale (SOMAS). Both items were scored on a scale from 1–5.

BMI, body mass index; SD, standard deviation.

### Relation Between Serum Inflammatory Markers and Measures of Motor Activity

When all patients were analyzed together, scores of increased motor activity were significantly negatively correlated with IFN-γ (rho = −0.128, p = 0.033, **Table 2**). In addition, we found a trend towards a positive correlation between TGF-β and increased motor activity (rho = 0.118, p = 0.050). No significant correlations where found between cytokines, motor retardation, and increased motor activity analyzing the two subgroups non-affective psychosis and unipolar depression (**Table 2**). In a multiple-linear-regression model correcting for age, gender, and BMI, the associations between increased motor activity and IFN-γ (beta = −0.112, t = −1.726, p = 0.086) and TGF-β (beta = 0.123, t = 1.904, p = 0.058) did not remain significant. No findings remained significant after correcting for multiple testing with the Bonferroni correction.

**Table 2:** Correlation coefficients (rho) between serum cytokines, motor retardation, and motor activity^a^.

	IL-1β	IL-6	TNF-α	IFN-γ	IL-10	IL-4	TGF-β
*All patients*							
-Motor retardation	0.112	0.066	−0.027	0.090	0.061	−0.043	−0.042
-Motor activity	0.001	−0.010	−0.034	−0.128*	−0.002	0.057	0.118
*Non-affective psychosis*							
-Motor retardation	0.154	0.165	0.002	0.191	0.194	−0.181	−0.153
-Motor activity	0.052	0.251	0.029	0.000	0.085	0.080	−0.054
*Unipolar depression*							
-Motor retardation	−0.089	−0.003	−0.047	−0.007	−0.056	0.059	0.173
-Motor activity	−0.046	−0.133	−0.181	−0.173	−0.239	−0.002	0.107

*p ≤ 0.05.

a Motor retardation and motor activity were scored on a scale from 1–5.

IFN, interferon; IL, interleukin; TGF, transforming growth factor; TNF, tumor necrosis factor.

### Comparisons of Cytokine Levels Based on the Presence or Absence of Increased Motor Activity and Motor Retardation

**Figure 1:**
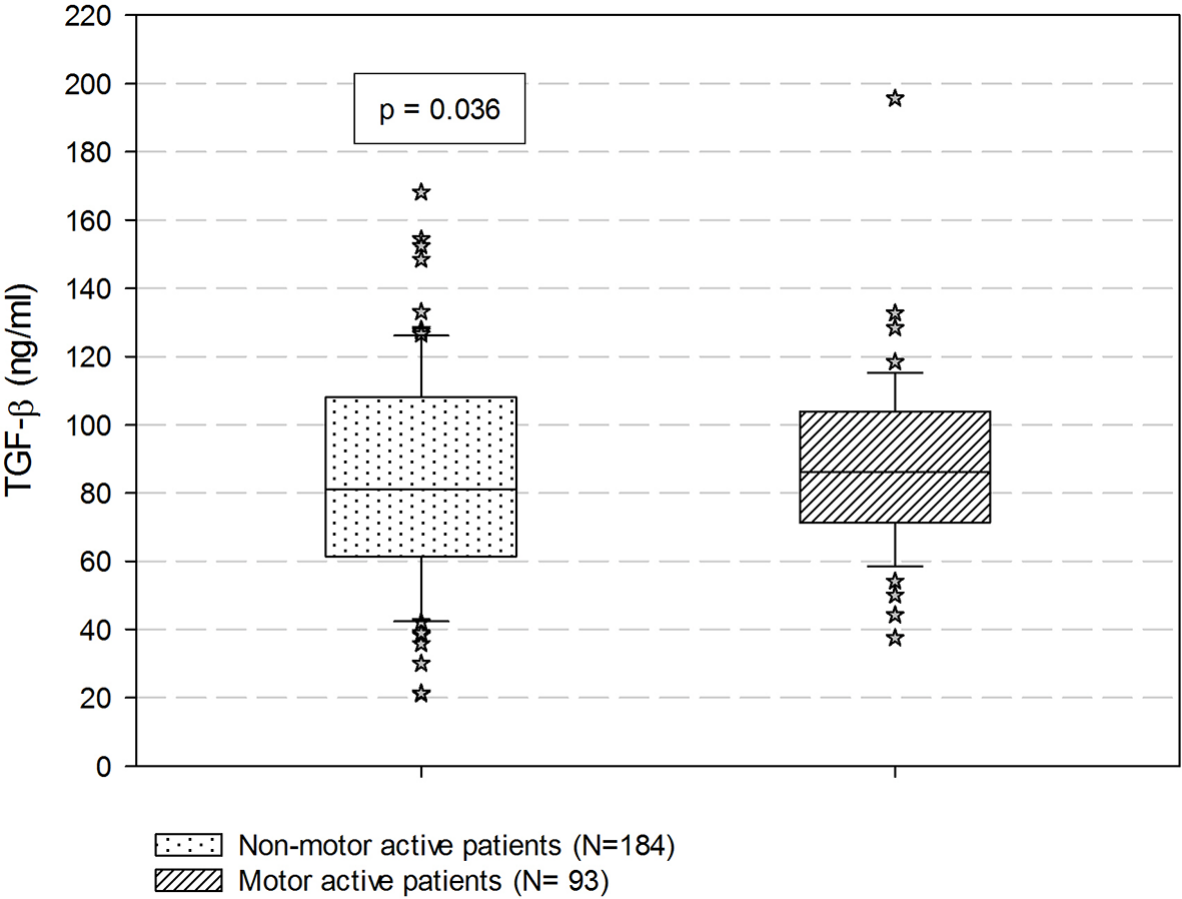
Comparisons of serum TGF-β based on the prevalence of increased motor activity. P-value is estimated by student’s independent samples t-test with log transformed values. Data are expressed as median with percentiles. TGF-β, transforming growth factor β.

**Figure 2:**
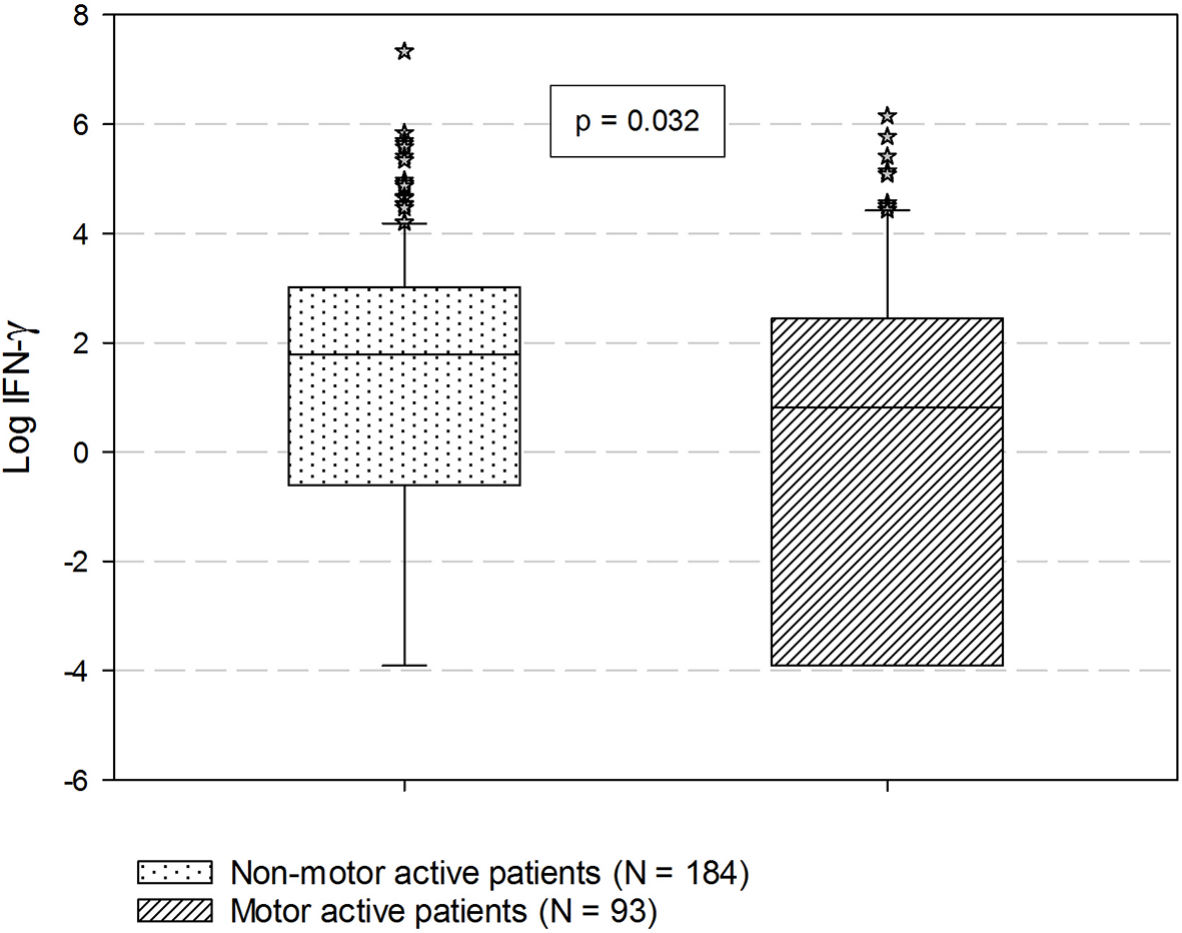
Comparisons of serum IFN-γ based on the prevalence of increased motor activity. P-value is estimated by Mann-Whitney U test. Data are expressed as median with percentiles. IFN-γ, Interferon-γ.

Mean log-transformed values of TGF-β were significantly higher in patients with increased motor activity compared to those with no increase in motor activity (11.31 ± 0.34 vs. 11.20 ± 0.45, t = −2.11, df = 236.41, ES = 0.016, p = 0.036) (**Figure 1**). The increased-motor-activity group had significantly lower levels of IFN-γ compared to the group without increased motor activity (2.27 ± 11.52 vs 6.00 ± 20.04, U = 7213, d = 0.13, p = 0.032) (**Figure 2**). These findings were however not significant after the Bonferroni correction for multiple testing. No other differences in cytokine levels between groups reached the level of significance. When comparing cytokine levels between the two groups with and without motor retardation, no statistical differences were detected (data not shown).

## Discussion

After correcting for multiple testing and confounders, we did not find any significant association at the 0.05-level between motor activity and cytokines. However, a trend towards an association between increased motor activity and lower serum levels of IFN-γ and higher levels of TGF-β in patients admitted to an acute psychiatric ward was seen. No statistically significant association between motor retardation and cytokines was seen. To our knowledge, this is the first report on the relation between immune markers and motor activity in an acute inpatient psychiatric population.

Aggression is a psychiatric sign associated with increased motor activity (15). In a recent study on inpatients with schizophrenia, aggressive behavior was associated with increased levels of Th17 cytokines TGF-β, IL-17, and IL-23 (16). In the present study, we examine motor activity only, but our finding of a trend towards increased TGF-β may be in line with the report on aggressive behavior. However, our findings did not remain significant after corrections for multiple testing and confounders. One might therefore also interpret this previous finding as not being in line with our study.

The present study did not show any significant difference in cytokine levels in relation to motor retardation. This may be somewhat surprising as other studies have indicated a relation between motor retardation and Th1 cytokines. Increased Th1 response has been demonstrated in association with low number of steps per day (17) as well as reduced psychomotor speed (11) in outpatients with major depression. Also, treatment with IFN-α increases the risk of depression and reduced psychomotor speed in patients with hepatitis C (18) and is associated with higher degree of motor retardation in depression (19). However, none of these studies were conducted in an acute psychiatric population, making our results not directly comparable. It is possible that other inflammatory factors are stronger in the acute population masking an association between Th1 and retardation.

There are several limitations and strengths to the current study. We did unfortunately not calculate power for this part of the study. The degree of motor retardation and increased motor activity was assessed by items from SOMAS, which is validated for this purpose. However, the items are published in a previous study (14, 20), and is also shown to correspond with findings in actigraphy (2). For future studies actigraphy should be included. Finally, even though alterations in cytokines in relation to motor activity were influenced by age, gender, and BMI, cytokines still may be clinically important.

Several of the serum cytokines had a high percentage of samples below the detection limit. Although this was not the case for IFN-γ and TGF-β, it may have affected the analyses of other pro-inflammatory cytokines, increasing the risk for type-II error. Further, the results for subgroup analyses are limited by a relatively low number of participants in the subgroups non-affective psychosis and unipolar depression and by the lack of a healthy control group. However, it is interesting as it suggests that alterations in immune activity are more related to symptoms than diagnostic group.

The study population was also relatively heterogeneous with the possibility of confounding factors. Thus, all findings need replication and should be interpreted with care.

We were however able to include severely ill patients in acute states with a variety of psychiatric diagnoses. The blood samples were drawn during the first 24 h of the admission, the period in which the symptoms were most prominent. In addition, the clinic recruiting the study participants is the only acute psychiatry inpatient service in the catchment area, reducing the effect of socioeconomics. All patients in the area needing acute psychiatric services were admitted to this unit. Also, our total sample size is relatively large, compared to other studies in the field.

## Conclusions

Our study comparing levels of immune markers in an acute setting did not reveal any significant associations between altered motor activity and cytokine levels. However, a trend towards a Th17 profile among patients with increased motor activity was seen. The finding should be further explored because it may have implications for predicting and treating deviations in motor activity.

## Data Availability Statement

All datasets generated for this study are included in the article/**Supplementary Material**.

## Ethics Statement

The studies involving human participants were reviewed and approved by Regional Committees for Medical and Health Research Ethics (REC) Central Regional, Trondheim, Norway. The patients/participants provided their written informed consent to participate in this study.

## Author Contributions

JL did all statistical analyses, took a leading role in selection of analyses and interpretation of the results, and wrote the original draft for the manuscript. AS did all the laboratory analyses on cytokines. AS took an equal part in planning and discussing the choice of analyses, interpretation of results and writing of the manuscript. VI supervised on statistics and took an equal part in choice and discussion of the statistical analyses as well as writing of the manuscript. AV initiated the study and headed the inclusion of patients in the clinic. AV took an equal part in interpretation of the results and writing of the manuscript. SR collaborated in initiation and performance of the study in the clinic. SR together with JL took a leading role in choice of laboratory analyses as well as interpretation of the data and in writing of the manuscript.

## Funding

This work was supported by the Norwegian University of Science and Technology, Department of Mental Health, St. Olav’s University Hospital, Division of Mental Health Care, and the Liaison Committee between the Central Norway Regional Health Authority. The funding organization had no role in the design, analysis, interpretation, or publication of the study.

## Conflict of Interest

The authors declare that the research was conducted in the absence of any commercial or financial relationships that could be construed as a potential conflict of interest.
